# Attitudes toward depression among Ecuadorian physicians using the Spanish-validated version of the Revised Depression Attitude Questionnaire (R-DAQ)

**DOI:** 10.1186/s40359-023-01072-y

**Published:** 2023-02-15

**Authors:** José Alejandro Valdevilla Figueira, Hans Mautong, Genesis Camacho L, Michelle Cherrez, Carlos Orellana Román, Geovanny Efraín Alvarado-Villa, Zouina Sarfraz, Azza Sarfraz, Arjola Agolli, María José Farfán Bajaña, Emanuel Vanegas, Miguel Felix, Jack Michel, Fernando Espinoza-Fuentes, Jaime Rosero Maquilón, Ivan Cherrez Ojeda

**Affiliations:** 1 Instituto de Neurociencias, Guayaquil, Ecuador; 2 Departamento de Psicología. Facultad de Marketing, Universidad Ecotec, Guayaquil, Ecuador; 3 Respiralab Research Group, Guayaquil, Ecuador; 4 Universidad Espíritu Santo, Km. 2.5 vía La Puntilla, 0901-952 Samborondón, Ecuador; 5 Universidad del Zulia, Maracaibo, Venezuela; 6 Research and Publications, Fatima Jinnah Medical University, Lahore, Pakistan; 7 Pediatrics and Child Health, The Aga Khan University, Karachi, Pakistan; 8 Division of Clinical and Translational Research, Larkin Healthcare System, Miami, USA; 9 MVZ für Entwicklungsförderung von Kindern GmbH / SBT, Berlin, Germany; 10 New York City Health + Hospitals, Woodhull, Brooklyn, NY USA; 11 New York City Health + Hospitals, Lincoln, Bronx, NY USA

**Keywords:** Attitudes, Depression, Physicians, Latin America, Stigmatization

## Abstract

**Purpose:**

Depression is inadequately recognized and managed, and physicians’ attitudes toward this condition and its treatment may play a part in this. This study aimed to assess Ecuadorian physicians' attitudes toward depression.

**Methods:**

This was a cross-sectional study conducted using the validated Revised Depression Attitude Questionnaire (R-DAQ). The questionnaire was delivered to Ecuadorian physicians, and the response rate was 88.8%.

**Results:**

76.4% of participants had never received previous training in depression, and 52.1% of them indicated neutral or limited professional confidence when dealing with depressed patients. More than two-thirds of the participants reported an optimistic attitude toward the generalist perspective of depression.

**Conclusion:**

Overall, physicians in Ecuador's healthcare settings were optimistic and held positive attitudes toward patients with depression. However, a lack of confidence in the management of depression and a need for ongoing training were found, especially among medical professionals who are not in daily contact with patients with depression.

**Supplementary Information:**

The online version contains supplementary material available at 10.1186/s40359-023-01072-y.

## Introduction

Mental illnesses are increasingly recognized as a public health concern. They account for 30% of the nonfatal disease burden and 10% of the global disease burden, including disability and death [1]. A combination of genetic, biological, environmental, and psychological factors appears to play a role in the disease [2]. In general, one out of every six adults will experience depression at some point in their lives [3].

Several sociodemographic factors are associated with the development of depression and help-seeking behaviors, such as educational level, female sex, and age at presentation[4]. Physicians’ characteristics could influence attitudes, responses, and treatment decisions toward depression. For instance, a study reported male participants to feel more confident about treating depression than female participants[5]. Camacho et al. found that Argentine male physicians have a higher tendency to think that depression is a normal part of aging[6], while Rojas Vistorte et al. found that primary care physicians with higher stigma are more reluctant to treat patients with mental illness, and thus have higher referrals to psychiatrists[7].

Depression is more common and more disabling in people with chronic medical conditions, such as type 2 diabetes, coronary heart disease, and asthma [8, 9]. Its presentation may differ among different population groups, and it is imperative to identify the risk factors, diagnose them early, and treat them appropriately. However, different cultural beliefs, interest in diseases, and, in some cases, stigmatization by physicians themselves limit the recognition of and attention to this condition.

Clinicians' attitudes toward depression may contribute to underdiagnosis and undertreatment of depression. In their study, Pelletier et al. found that an estimated 5.4% (1.5 million) of Canadians aged 15 years and older had symptoms consistent with a mood disorder, but only half had been professionally diagnosed [10]. To address the underdiagnosis of depression, Sapag et al. suggest that there is an urgent need to understand and reduce stigma in mental health services in primary health care settings [11]. A review of studies of stigma and health professionals indicated that both health professionals and the general public hold critical and stigmatizing views to varying degrees, nearly three-quarters of the relevant publications report that mental health providers' beliefs are no different from those of the general population or are even more negative [12].

Doctors are not immune to prejudices and stigmatizing attitudes toward people with mental illnesses [13]. For instance, a previous systematic review found that people with mental illnesses and substance abuse disorders received lower-quality treatment for a variety of physical illnesses [14]. Examining and assessing physicians' attitudes toward depression could provide insight into specific educational activities or large campaign initiatives and advisory for better understanding and systematic evaluation of depression in clinical practice. In the long run, these interventions will improve the recognition and management of depression, enhancing patient care quality among non-mental health physicians [15].

This study aimed to assess the attitudes of a sample of Ecuadorian physicians toward depression by using the validated Spanish Revised Depression Attitude Questionnaire (R-DAQ). The study also serves as the foundation for assessing the cultural values of the region associated with this mental illness and the strategies to be implemented to improve the quality of care for patients, including medical education, periodic evaluations, and new public policies to prevent stigmas that may restrict patient care. Finally, we also explored the relationship between participants’ attitudes with their demographic characteristics and training.

## Methods

### Study design and population

This cross-sectional study was conducted using the validated Spanish Revised Depression Attitude Questionnaire (R-DAQ) [16]. We used a convenience sampling technique to distribute the questionnaire. Practicing physicians from private and public services were eligible for the study. Participants were recruited from different hospitals, outpatient clinics, and private practices across Ecuador. The questionnaire was delivered to 564 Ecuadorian physicians; however, 63 professionals declined to participate in the study. A total of 501 participants completed the survey, with a response rate of 88.8%.

### Data collection and instrument

A self-report survey that included questions about the participants' demographic characteristics and medical training, along with the R-DAQ scale, was used. The Spanish translation of the R-DAQ (C_α_ = 0.78) consists of a 22-item scale divided into three subscales or factors, namely (1) Factor 1 (C_α_ = 0.80): “*Professional confidence regarding depression management*”, (2) Factor 2 (C_α_ = 0.71): “*Therapeutic optimism regarding depression treatment*” and (3) Factor 3 (C_α_ = 0.61): “*Generalist perspective of the occurrence, recognition, and management of depression*”. Each item is measured on a 5-point (1–5) Likert-type scale (ranging from “*strongly disagree*” = 1 to “*strongly agree*” = 5). Factor 1 (*Professional confidence regarding depression management*) consists of seven items (I-1, I-7, I-8, I-11, I-15, I-17 and I-19) that measure participants' comfort with and confidence in managing depressed patients. Factor 2 (*Therapeutic optimism regarding depression treatment*) is composed of 10 negative-stated items (I-3, I-4, I-5, I-6, I-9, I-12, I-13, I-18, I-20 and I-21) regarding the treatment of depression. Finally, Factor 3 (*Generalist perspective of the occurrence, recognition, and management of depression*) consists of five items (I-2, I-10, I-14, I-16 and I-22) that measure views of the occurrence, recognition, and management of depression. The scale meets the psychometric requirements for measuring attitudes toward depression in a Spanish-speaking population; it also has demonstrated face, content, and construct validity, as well as adequate internal consistency and test–retest reliability (Cronbach’s α of 0.8) [16].

### Procedure

A convenience sampling technique was applied. A qualified and previously trained physician approached professionals at hospitals, outpatient clinics, and private practices. After information about the study was provided and its objective was explained, informed consent to participate was obtained. Then, the trained personnel gave the participants a printed copy of the survey to complete. Participants could fill out their questionnaires by themselves by following the instructions provided or could obtain help from the trained personnel.

### Statistical analysis

All statistical analyses were performed using SPSS for Windows (version 24.0; SPSS, Inc., Chicago, Illinois). The Kolmogorov–Smirnov test was used to determine the normality of distribution. Categorical variables are presented as percentages. Continuous variables are presented as mean and standard deviation if normally distributed, otherwise they are displayed as medians and interquartile ranges.

For descriptive analyses, the categories of agreement and disagreement were grouped in a dichotomic fashion to reflect a positive attitude toward depression. For instance, for positive-stated items (I-1, I-2, I-7, I-10, I-11, I-14, I-15, I-16, I-17, I-19 and I-22) the categories “*agree*” and “*strongly agree*” were grouped under one category while the subgroups “*strongly disagree*”, “*disagree*” and “*neutral*” were included under another designation. Conversely, for negative-stated items (I-3, I-4, I-5, I-6, I-8, I-9, I-12, I-13, I-18, I-20 and I-21) the “*strongly disagree*” and “*disagree*” categories were consolidated under one group as it would denote a positive attitude towards depression, while the remainder (“*neutral*”, “*agree*” and “*strongly agree*”) categories were grouped in a separate one.

Multiple linear regression models were used to predict the overall R-DAQ score and each of its factors from age, gender, specialty, type of service, area of service, frequency of depression consult and previous depression training. However, to perform such analyses a series of statistical assumptions were explored after which the statistical significance of each model was evaluated to determine how well each of them fit. As such, the independence of observations was checked through the Durbin-Watson statistic. Linearity was assessed for each variable collectively and individually by plotting a scatterplot of the studentized residuals and partial regression plots, respectively. Also, the same plots of studentized residuals against the unstandardized predicted values were used to assess homoscedasticity. Then, multicollinearity was ruled out through Tolerance (cut-off value of 0.1) or/and VIF (cut-off value of 10) values; values above the cut-off were considered to meet the assumption. While outliers were evaluated through casewise diagnostics and studentized deleted residuals, high leverage or influential points were identified using Cook’s distance as a measure. Finally, either Q-Q plots of the studentized residuals or histograms with superimposed normal curves and a P-P plot were used to check for the normal distribution of residuals (errors of prediction). Statistical significance was defined as a 2-tailed *p* < 0.05.

### Statistical power

We calculated statistical power using G*Power Version 3.1.9.4. We set a medium effect size of 0.15, the α error probability of 0.05, 7 predictor variables, and a sample size of 501. Considering such parameters, the computed power was 1.00.

### Ethical considerations

This study was conducted in accordance with the Declaration of Helsinki and was approved by the ethics committee: Comité de ética e Investigación en Seres Humanos (CEISH), Guayaquil-Ecuador (#HCK-CEISH-18-0060). Informed consent was obtained from all participants before their voluntary participation in the survey. The participants could not be personally identified using the information recollected in the survey; thus, their anonymity was preserved, and their personal data were protected.

## Results

### Demographic characteristics of the study population

The demographic characteristics of the study population are presented in Table 1. A total of 501 physicians completed the survey. Their mean age was 49.0 years (SD, 11.3). The female-to-male ratio was nearly 1:1. More than half (53.3%, n = 267) were primary care physicians; 8.0% (n = 40) were psychiatrists whilst the remainder (38.7%, n = 194) belonged to other specialties such as family medicine, cardiology, neurology, pediatrics, among others. Most of these physicians worked in an urban setting (83.6%, n = 419), and more than half worked exclusively in their private practice (48.7%, n = 244). Furthermore, 76.4% (n = 382) of the participants had never received previous training in depression through medical education courses, and 40.1% (n = 201) of them never or rarely had depression consultations in their practice.

**Table 1:** Demographic characteristics of selected participants (n = 501)

Variable	% (n)
Age (mean, SD)	49.0 (11.3)
Gender	
Male	49.5 (248)
Female	49.9 (250)
Specialty	
Primary care	53.3 (267)
Psychiatry	8.0 (40)
Other	38.7 (194)
Service type	
Private	48.7 (244)
Public	40.3 (202)
Public and private	10.4 (52)
Service area	
Urban	83.6 (419)
Rural	9.0 (45)
Urban and rural	1.0 (5)
Frequency of depression consults	
Never	8.4 (42)
Rarely	31.7 (159)
Sometimes	32.7 (164)
Often	16.4 (82)
Frequently	10.6 (53)
Previous depression training	
Yes	23.4 (117)
No	76.4 (382)
R-DAQ score (media, IQR)	76.0 (13)
Factor 1* (median, IQR)	22.0 (7)
Factor 2** (median, IQR)	35.0 (7)
Factor 3*** (median, IQR)	20.0 (5)

*Factor 1: “Professional confidence regarding depression management”

**Factor 2: “Therapeutic optimism regarding depression treatment”

***Factor 3: “Generalist perspective of the occurrence, recognition, and management of depression”

### Attitudes toward depression

The frequency of responses reflecting a positive attitude from participants towards depression is included in Additional file 1: Table S1. Overall, the participants showed a slightly positive attitude towards depression, with a median score of 76 (IQR, 13). The median score of each independent factor and their interquartile range are listed in Table 1.

The respondents’ professional confidence was neutral or limited; most of them indicated that they were comfortable dealing with depressed patients’ needs (52.1%, n = 261) and believed that their profession was well-positioned to help depressed patients (50.9, n = 255). However, less than half of the respondents believed that their profession was well-trained to help patients with depression (42.3%, n = 212) or felt confident in assessing depression (43.9%, n = 220) or suicidal risk in depressive patients (41.3%, n = 207).

Most of the participants indicated therapeutic optimism regarding depression; most participants disagreed that suicidal thinking was unstoppable (70.5%, n = 353) or that psychological (64.1%, n = 321) and antidepressant (71.1%, n = 356) therapies are ineffective for the management of depression. However, 29.3% (n = 147) of them disagreed with the view that depression was not amenable to change. Finally, the participants appeared to endorse a generalist perspective about depression and its management, with more than two-thirds of participants agreeing with all the items of this factor excluding item 2. Most of the participants agreed that anyone can suffer from depression (78.4%, n = 393) and that all physicians should have the skills to recognize and manage depression (81.8%, n = 410).

### Linear regression of the overall R-DAQ and its subscales

A set of 4 multiple regression analysis models were run to predict the score of Factor 1 (Professional confidence regarding depression management), Factor 2 (Therapeutic optimism regarding depression treatment), Factor 3 (Generalist perspective of the occurrence, recognition, and management of depression), and R-DAQ from participants' age, gender, specialty, service type, service area, frequency of depression consults and previous depression training. All 4 models met the assumptions noted in the methods section and statistically significantly predicted the dependent continuous variable.

Regarding Factor 1 (Professional confidence regarding depression management), linearity was confirmed through visual inspection of partial regression plots and a plot of studentized residuals against predicted values. The independence of observations was determined by a Durbin-Watson statistic of 1.752. Homoscedasticity was assessed by visualization of studentized residuals versus unstandardized predicted values. No evidence of multicollinearity was evidenced through tolerance values greater than 1 (Table 2). No studentized deleted residuals greater than 3 SD, leverage values greater than 0.2, or value’s for Cook distance above 1 were found. The assumption of normality of residuals was met as assessed by Q-Q plots. The multiple regression model statistically significantly predicted the Factor 1 score, F (10, 450) = 15.989, *p* = 0.000, adj. R^2^ = 0.25. The analysis showed that being a primary care physician (*B*, 1.313) or being a psychiatrist (*B*, 4.469) predicted a higher score relative to being from another specialty. The same observation was recorded for participants with a higher frequency of depression consults (*B*, 1.450) and previous depression training (*B*, 1.678).

**Table 2:** Influence of participants' characteristics on their professional confidence and therapeutic optimism on depression

Variable	Factor 1				Tolerance	Factor 2				Tolerance
	Sig	*B*	95% CI for *B*			Sig	*B*	95% CI for *B*		
			LL	UL				LL	UL	
Age	.289	− .021	− .061	.018	.895	.376	− .022	− .070	.026	.895
Gender^a^										
Female	.640	− .210	− 1.089	.669	.961	.007	1.465	.404	2.527	.961
Specialty^b^										
Primary care	.008	1.313	.343	2.282	.791	.766	.177	− .993	1.348	.791
Psychiatry	.000	4.469	2.564	6.374	.659	.001	3.897	1.596	6.197	.659
Service type^c^										
Private	.295	− .512	− 1.472	.448	.805	.001	1.952	.793	3.111	.805
Public and private	.931	− .068	− 1.625	1.488	.851	.028	2.106	.227	3.986	.851
Service area^d^										
Urban	.285	.803	− .671	2.276	.882	.723	− .321	− 2.100	1.458	.882
Urban and rural	.991	.026	− 4.402	4.455	.880	.670	1.161	− 4.187	6.508	.880
Frequency of depression consults	.000	1.450	.995	1.906	.705	.053	.544	− .006	1.094	.705
Previous depression training^e^										
Yes	.006	1.678	.490	2.865	.723	.562	.424	− 1.011	1.858	.723

Regression analyses were adjusted for variables such as age, gender, specialty, service type, service area, frequency of depression consults and previous depression training. Values are significant at .05 significance level. *B*, unstandardized regression coefficient; CI, confidence interval; LL, lower limit; UL, upper limit

^a^Reference *gender* category is "male"

^b^Reference *specialty* category is "other"

^c^Reference *service type* category is "public"

^d^Reference *service area* category is "rural"

^e^Reference *previous depression training* is "No"

For Factor 2 (Therapeutic optimism regarding depression treatment), through visual inspection of partial regression plots and a plot of studentized residuals against predicted values, linearity could be assessed. A Durbin-Watson statistic of 1.850 determined the independence of observations. Visualization of studentized residuals versus unstandardized predicted values showed homoscedasticity. Multicollinearity was ruled out through tolerance values greater than 1 (Table 2). No studentized deleted residuals greater than 3 SD, leverage values greater than 0.2, or value’s for Cook distance above 1 were found. Q–Q plots revealed the normal distribution of residuals. The multiple regression model statistically significantly predicted the Factor 2 score, F (10, 450) = 6.316, *p* = 0.000, adj. R^2^ = 0.10. It was found that being a female (*B*, 1.465) as well as specializing in psychiatry (*B*, 3.897) predicted a higher score. Also, working in the private (*B*, 1.952) or private/public (*B*, 2.106) service was statistically significantly associated with a higher Factor 2 score concerning those providing exclusively public service.

Meanwhile, for Factor 3(Generalist perspective of the occurrence, recognition, and management of depression), linearity was assessed by visualizing partial regression plots and a plot of studentized residuals against predicted values. Independence of observations was established through a Durbin-Watson statistic of 1.753. Plots of studentized residuals versus unstandardized predicted values were compatible with homoscedasticity. No multicollinearity could be found as tolerance values were greater than 1 (Table 3). No studentized deleted residuals greater than 3 SD, leverage values greater than 0.2, or value’s for Cook distance above 1 were found. The normality of residuals was witnessed through Q–Q plots. The multiple regression model statistically significantly predicted the Factor 3 score, F (10,450) = 3.122 *p* = 0.000, adj. R^2^ = 0.04. In this model, it was determined that an older age predicted a lower score (*B*, − 0.034). However, providing private/public service (*B*, 2.079) and having a higher frequency of depression consults (*B*, 0.535) predicted the opposite.

**Table 3:** Influence of participants' characteristics on the generalist perspective about the occurrence, recognition and management of depression and R-DAQ overall score

Variable	Factor 3				Tolerance	Overall score				Tolerance
	Sig	*B*	95% CI for *B*			Sig	*B*	95% CI for *B*		
			LL	UL				LL	UL	
Age	.027	− .034	− .064	− .004	.895	.044	− .077	− .152	− .002	.895
Gender^a^										
Female	.855	.062	− .601	.724	.961	.119	1.317	− .341	2.975	.961
Specialty^b^										
Primary care	.424	.298	− .433	1.029	.791	.055	1.788	− .041	3.616	.791
Psychiatry	.787	− .198	− 1.634	1.239	.659	.000	8.168	4.575	11.761	.659
Service type^c^										
Private	.426	.293	− .430	1.017	.805	.060	1.734	− .076	3.544	.805
Public and private	.001	2.079	.905	3.253	.851	.006	4.117	1.181	7.053	.851
Service area^d^										
Urban	.355	.523	− .588	1.634	.882	.478	1.005	− 1.774	3.784	.882
Urban and rural	.786	.461	− 2.878	3.801	.880	.698	1.648	− 6.704	10.001	.880
Frequency of depression consults	.002	.535	.191	.878	.705	.000	2.529	1.670	3.388	.705
Previous depression training^e^										
Yes	.751	− .145	− 1.041	.751	.723	.087	1.956	− .284	4.196	.723

Regression analyses were adjusted for variables such as age, gender, specialty, service type, service area, frequency of depression consults and previous depression training. Values are significant at .05 significance level. *B* unstandardized regression coefficient, *CI* confidence interval, *LL* lower limit, *UL* upper limit

^a^Reference *gender* category is "male"

^b^Reference *specialty* category is "other"

^c^Reference *service type* category is "public"

^d^Reference *service area* category is "rural"

^e^Reference *previous depression training* is "No"

Finally, for the R-DAQ overall score, linearity was confirmed by visual inspection of partial regression plots and a plot of studentized residuals against predicted values. A Durbin-Watson statistic of 1.921 verified the independence of observations. Homoscedasticity was determined through plots of studentized residuals versus unstandardized predicted values. Tolerance values greater than 1 (Table 3) denied multicollinearity. No studentized deleted residuals greater than 3 SD, leverage values greater than 0.2, or value’s for Cook distance above 1 were found. Q–Q plots were compatible with the normal distribution of residuals. The multiple regression model statistically significantly predicted the overall R-DAQ score, F (10,450) = 15.001 *p* = 0.000, adj. R^2^ = 0.23. Coefficients showed that while an older age predicted a lower R-DAQ overall score (*B*, − 0.077), being a psychiatrist (*B*, 8.168), providing public/private services (*B*, 4.117) and a higher volume of depression consults (*B*, 2.529) were associated with a higher R-DAQ overall score.

## Discussion

In our study, we found most physicians did not have any type of training in depression; 76.4% of respondents agreed that they had not taken any type of continuing medical education that focused on depression, even though nearly half of them felt they had the necessary skills to assist depression patients. In contrast, Norton et al. reported that 80% of participants thought they needed training to handle depressed patients, as they were insufficiently trained [17]. This is a crucial point since without mental health training for physicians it will be almost inefficient to promote universal health and appropriate treatment to reduce mental health problems [18].

Despite the lack of training, half the respondents (50.9%; Additional file 1: Table S1) were optimistic about their profession's potential to assist patients with depression similarly reported in a study made in Pakistan (54.8%). Pakistanis felt more comfortable working with physical rather than mental health problems (59.2%) in a greater percentage, in contrast to our study, where only 25% agreed with this statement [19]. The UK and European nations have generally a more optimistic attitude toward depression, and physicians tend to be more confident than Americans. A pooled analysis of DAQ in 2012 reported less stigmatization in Europe countries (32.9% in the UK, 37.7% in France and Italy) than in our study, where 69.9% of respondents believed depression is just part of aging, and 50.7% agreed that it is a way for people with low stamina to cope with life [20].

Aldahmashi et al. reported that the majority of the medical professionals in their study preferred dealing with physical rather than mental illness [21]. In Latin America, Camacho-Leon et al. reported that 74.6% of physicians from three countries (Argentina, Chile, and Venezuela) agreed they felt more at ease treating physical ailments (70.3%). About 74.3% of Ecuadorian physicians answered similarly, reflecting a similar pattern among the countries of the region. The previous data reinforce the idea that understanding the attitudes of medical practitioners is important for shaping service delivery and assessing training needs [21].

Aldamashi et al. indicated that psychological therapy tends to be unsuccessful with people who are depressed (12.4%) and that once a person has made up their mind about taking their own life, no one can stop them (8.7%). In fact, the percentage of Ecuadorian physicians who held these views was quite high; participants reported believing psychological therapy to be ineffective (35.9%) and that suicidal ideation was unstoppable (29.3%) were much higher than the percentage of Saudi participants who agreed with these statements. [21] This might be related to the fact that most of the respondents in the present study had never received training (76.4%), and never or rarely had been consulted about depression in their practice (40.1%), also, the majority admitted that they did not feel comfortable assessing depression (43.9%) or suicidal risk (41.3%). As Richards et al. shows, mental health training in the previous five years has a positive effect on general attitudes toward depression, whereas a lack of such training and experience may adversely affect the performance and attitudes of healthcare providers [22]. Nevertheless, some respondents agreed depression has care needs like other conditions (65.7%), consider it an important part of managing other health problems (78.2%), and also that physicians should have skills in recognizing and managing depression (81.8%). Our results are similar to those reported in other studies; however, a smaller percentage (62.9%) reported thinking that depression is like any other disease. [21]

According to a 2018 study despite their tendency to treat depression, primary care physicians prefer to refer patients to psychiatrists[7], nonetheless, scores obtained in personal confidence regarding depression by primary care physicians (B, 1.313) and psychiatrists (B, 4.469) were higher than that the achieved by other professionals. Furthermore, it was found that being female (B, 1.465) as well as having a specialty in psychiatry (B, 3.897) predicted higher optimism regarding depression. A study found that women physicians provide better medical care than their male counterparts [23]. Additionally, those who work in the private sector (B, 1.952), or in the private/public sector (B, 2.106), have higher therapeutic optimism regarding depression than those who work exclusively in the public sector. According to researchers, dissatisfaction with income levels affects how physicians feel and what they recommend to their patients daily [24].

Physicians with previous depression training and frequent depression consultations had greater professional confidence and overall R-DAQ scores as well as a broader perspective and greater therapeutic optimism, according to our results and other studies[5, 17]. Based on the evidence, doctors' optimistic views and confidence are related to their patients' success in healing through the placebo effect[25].

This study may provide a better understanding of the treatment of mental illnesses in Ecuador, while also laying the groundwork for future projects that will foster a better attitude toward depression among physicians in the region by using its findings to implement strategies to improve patient care.

### Limitations

The participants were recruited from Ecuador, and physicians with psychiatric and nonpsychiatric backgrounds were part of the study. Some limitations were present in this study, principally because the attitudes of pediatricians toward depression were not assessed, which made it impossible to analyze the management of depression in children. Another important point that was not applied in this study was the presence of cultural beliefs that might change physicians’ attitudes; this omission restricted the analysis and limited comparisons with similar studies that did consider such beliefs. Attitudes toward the diagnosis of depression in atypical presentations were not evaluated, and neither was the diagnosis of depression in the presence of addictions, both of which have been described as generally underdiagnosed in previous studies[26, 27]. Also, convenience sampling could have led to Type I error as participants may have not accurately portrayed the population we described; thus, results should be extrapolated cautiously.

## Conclusion

Overall, physicians in Ecuador's healthcare settings were optimistic and held positive attitudes toward patients with depression. However, a lack of confidence in the management of depression and the need for continuous training were found, especially in medical professionals who are not in daily contact with patients with depression. Further research is necessary to better understand how physicians’ training can be improved since knowledge and perception of depression may impact attitudes, responses, and treatment decisions made for patient care.

## Supplementary Information

**Additional file 1: Table S1.** Frequency of responses to R-DAQ items reflecting a positive attitude towards depression by factor.

## Data Availability

The datasets used and/or analyzed during the current study are available from the corresponding author upon reasonable request.

## Abbreviation

R-DAQ: Spanish validated version of the Revised Depression Attitude Questionnaire
