# Supplementary material for: Attitudes toward depression among Ecuadorian physicians using the Spanish-validated version of the Revised Depression Attitude Questionnaire (R-DAQ)
Source: BMC Psychol. 2023 Feb 15;11:46. doi: 10.1186/s40359-023-01072-y (PMC9930300; doi:10.1186/s40359-023-01072-y)
Supplement: Supplementary file 1 — Additional file 1: Table S1. Frequency of responses to R-DAQ items reflecting a positive attitude towards depression by factor. [file 40359_2023_1072_MOESM1_ESM.docx]

| Table S1 - Frequency of responses to R-DAQ items reflecting a positive attitude towards depression by factor | |
| --- | --- |
| **R-DAQ Item** | % (n) |
| **Factor 1 - *Professional confidence regarding depression*** |  |
| I-1. *I feel comfortable dealing with depressed patients’ needs*^a^ | 52.1 (261) |
| I-7. *I feel confident in assessing depression in patients*^a^ | 43.9 (220) |
| I-8. *I am more comfortable working with physical illness than with mental illnesses like depression^b^* | 25.7 (129) |
| I-11. *My profession is well positioned to assist patients with depression^a^* | 50.9 (255) |
| I-15. *My profession is well trained to assist patients with depression*^a^ | 42.3 (212) |
| I-17. *I feel confident in assessing suicide risk in patients presenting^a^* | 41.3 (207) |
| I-19. *It is rewarding to spend time looking after depressed patients*^a^ | 45.1 (226) |
| **Factor 2 *- Therapeutic optimism regarding depression*** |  |
| I-3. *Psychological therapy tends to be unsuccessful for people who are depressed^b^* | 64.1 (321) |
| I-4. *Antidepressant therapy tends to be unsuccessful in people who are depressed^b^* | 71.1 (356) |
| I-5. *One of the main causes of depression is a lack of self-discipline and willpower^b^* | 42.5 (213) |
| I-6. *Depression treatments medicalize unhappiness^b^* | 37.3 (187) |
| I-9. *Being depressed is a natural part of being old^b^* | 69.9 (350) |
| I-12. *Being depressed is a way that people with poor stamina deal with life difficulties^b^* | 50.7 (254) |
| I-13. *Once a person has made up their mind to take their own life, no one can stop them^b^* | 70.5 (353) |
| I-18. *Depression reflects a response that is not amenable to change^b^* | 29.3 (147) |
| I-20. *Being depressed is a natural part of adolescence^b^* | 67.5 (338) |
| I-21. *There is little to be offered to depressed patients who do not respond to initial treatments^b^* | 59.7 (299) |
| **Factor 3 - *Generalist perspective about the occurrence, recognition, and management of depression*** |  |
| I-2. *Depression is a disease like any other (e.g., asthma, diabetes)*^a^ | 62.9 (315) |
| I-10. *All health professionals should have skills in recognizing and managing depression*^a^ | 81.8 (410) |
| I-14. *People with depression have care needs similar to other medical conditions like diabetes, COPD, or arthritis*^a^ | 65.7 (329) |
| I-16. *Recognizing and managing depression is often an important part of managing other health problems*^a^ | 78.2 (392) |
| I-22. *Anyone can suffer from depression*^a^ | 78.4 (393) |
| Notes: Frequencies are presented as percentages. Only responses reflecting positive attitudes towards depression are reported. ^a.^ Positive statements report categories of agreement ("agree" and "strongly agree") ^b.^ Negative statements report categories of disagreement ("*strongly disagree*" and "*disagree*") | |
